# Identification of Robust and Key Differentially Expressed Genes during C2C12 Cell Myogenesis Based on Multiomics Data

**DOI:** 10.3390/ijms23116002

**Published:** 2022-05-26

**Authors:** Song Zhang, Yuanyuan Zhang, Choulin Chen, Qingqing Hu, Yang Fu, Lingna Xu, Chao Wang, Yuwen Liu

**Affiliations:** 1 Shenzhen Branch, Guangdong Laboratory for Lingnan Modern Agriculture, Key Laboratory of Livestock and Poultry Multi-Omics of MARA, Agricultural Genomics Institute at Shenzhen, Chinese Academy of Agricultural Sciences, Shenzhen 518124, China; zhangsong@caas.cn (S.Z.); zhangyuanyuan02@caas.cn (Y.Z.); chenchoulin3456@webmail.hzau.edu.cn (C.C.); huqingqing@webmail.hzau.edu.cn (Q.H.); fuyang@caas.cn (Y.F.); xulingna@caas.cn (L.X.); wangchao2020@webmail.hzau.edu.cn (C.W.); 2 Innovation Group of Pig Genome Design and Breeding, Research Centre for Animal Genome, Agricultural Genomics Institute at Shenzhen, Chinese Academy of Agricultural Sciences, Shenzhen 518124, China; 3 School of Life Sciences, Henan University, Kaifeng 475004, China; 4 Key Laboratory of Agricultural Animal Genetics, Breeding and Reproduction of Ministry of Education and Key Laboratory of Swine Genetics and Breeding of Ministry of Agriculture, College of Animal Science and Technology, Huazhong Agricultural University, Wuhan 430070, China; 5 Kunpeng Institute of Modern Agriculture at Foshan, Chinese Academy of Agricultural Sciences, Foshan 528226, China

**Keywords:** myogenesis, differentially expressed genes, robust rank aggregation, KLF5, enhancer

## Abstract

Myogenesis is a central step in prenatal myofiber formation, postnatal myofiber hypertrophy, and muscle damage repair in adulthood. RNA-Seq technology has greatly helped reveal the molecular mechanism of myogenesis, but batch effects in different experiments inevitably lead to misinterpretation of differentially expressed genes (DEGs). We previously applied the robust rank aggregation (RRA) method to effectively circumvent batch effects across multiple RNA-Seq datasets from 3T3-L1 cells. Here, we also used the RRA method to integrate nine RNA-Seq datasets from C2C12 cells and obtained 3140 robust DEGs between myoblasts and myotubes, which were then validated with array expression profiles and H3K27ac signals. The upregulated robust DEGs were highly enriched in gene ontology (GO) terms related to muscle cell differentiation and development. Considering that the cooperative binding of transcription factors (TFs) to enhancers to regulate downstream gene expression is a classical epigenetic mechanism, differentially expressed TFs (DETFs) were screened, and potential novel myogenic factors (MAF, BCL6, and ESR1) with high connection degree in protein–protein interaction (PPI) network were presented. Moreover, KLF5 cooperatively binds with the three key myogenic factors (MYOD, MYOG, and MEF2D) in C2C12 cells. Motif analysis speculates that the binding of MYOD and MYOG is KLF5-independent, while MEF2D is KLF5-dependent. It was revealed that KLF5-binding sites could be exploited to filter redundant MYOD-, MYOG-, and MEF2D-binding sites to focus on key enhancers for myogenesis. Further functional annotation of KLF5-binding sites suggested that KLF5 may regulate myogenesis through the PI3K-AKt signaling pathway, Rap1 signaling pathway, and the Hippo signaling pathway. In general, our study provides a wealth of untapped candidate targets for myogenesis and contributes new insights into the core regulatory mechanisms of myogenesis relying on KLF5-binding signal.

## 1. Introduction

Skeletal muscle accounts for almost 40% of adult body weight, maintains posture and the balance of body metabolism [1,2], and is basically composed of myofibers. Myoblasts (MBs) undergo myogenesis to develop into myotubes (MTs), which further fuse to form myofibers. In the past few decades, the identification of key genes for myogenic differentiation has been a major research focus. Among these genes, the well-known myogenic regulatory factor (MRF) family (MYOD, MYOG, MYF5, and MRF4, also called MYF6) and myocyte enhancer factor 2 (MEF2) family (MEF2A, MEF2B, MEF2C, and MEF2D) genes play a crucial role in the process of myogenesis, acting synergistically to stimulate and initiate the differentiation process of MBs and induce the expression of several waves of muscle-specific genes by targeting their regulatory sequences, such as enhancers [3,4,5,6,7]. With further exploration, required and positive regulatory factors for myogenesis, such as KLF5 [8], STAT3 [9], and NR4A1 [10], have been continuously revealed in recent years. Currently, the understanding of the mechanisms of myogenesis remains limited, so methods for efficiently identifying key genes for myogenic differentiation are desired.

Using high-throughput methods (such as expression arrays and RNA-seq) to find differentially expressed genes (DEGs) before and after myoblast differentiation is a highly effective approach for identifying key regulatory factors for myogenesis. The array approach based on nucleic acid hybridization theory is a traditional technique for obtaining gene expression profiles and was widely used in early myogenesis research [11,12,13,14]. After nearly a decade, RNA-seq has gradually become the most widely applied method of gene expression profiling because of its multiple advantages, including the identification of low-abundance transcripts, splice variants, and novel coding and noncoding transcripts [15,16,17,18,19]. Naturally, the gene expression profiles of the myogenesis process based on RNA-seq showed an explosive increase [20,21,22,23,24,25,26,27,28]. The NCBI Gene Expression Omnibus (GEO) repository stores massive myogenesis gene expression datasets from different research projects. However, due to the existence of systematic errors, including differences in myogenic differentiation induction methods, cell batch effects, high-throughput platforms, and experimental operators, the change trend of DEG expression is often confusing.

**Figure 1:**
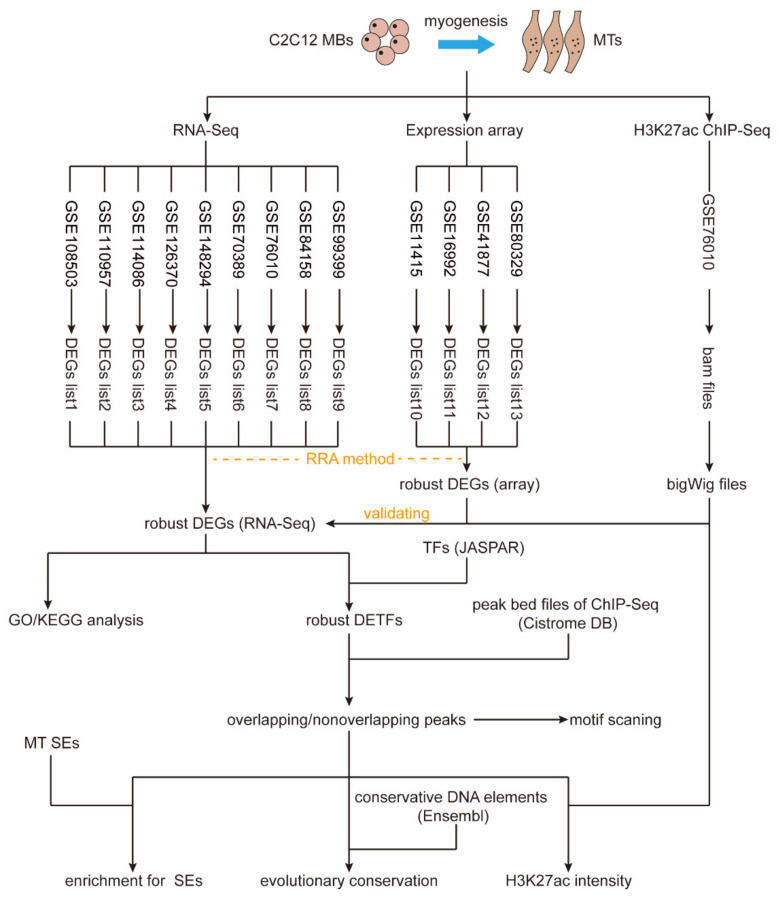
Study roadmap. Data processing and DEG identification were performed on 9 RNA-Seq datasets and 4 expression array datasets from C2C12 MBs and MTs, respectively. Nine DEG lists from RNA-Seq data and 4 DEG lists from array data were then integrated using the RRA method to obtain robust DEGs. In addition, the H3K27ac ChIP-Seq dataset was processed to obtain bigWig files representing the H3K27ac intensity in MBs and MTs. The accuracy of robust DEGs (RNA-Seq) was validated with robust DEGs (array) and H3K27ac intensity. If not specified, robust DEGs represent robust DEGs (RNA-Seq). Next, enrichment analysis for Gene Ontology (GO)/Kyoto Encyclopedia of Genes and Genomes (KEGG) terms was performed on robust DEGs. The list of vertebrate TFs was downloaded from the JASPAR database and intersected with the robust DEGs to obtain robust DETFs. The peak bed files of TF ChIP-Seq data were downloaded from the Cistrome Data Browser (DB), which were then processed to investigate the cooperative binding of robust DETFs. Finally, the motifs of overlapping and nonoverlapping target peaks are scanned, and the ability of these peaks to enrich H3K27ac signals, SEs, and conservative DNA elements was explored.

The robust rank aggregation (RRA) method is essentially a powerful sorting algorithm that generates reliable gene sorting results by integrating multiple sorted gene lists [29,30]. We previously applied the RRA approach to identify robust DEGs for 3T3-L1 cell differentiation by integrating multiple datasets [31]. The same approach was also performed in prostate cancer [32]. C2C12 cells derived from murine skeletal muscle cells are a well-established model for studying muscle regeneration and differentiation [33]. This study comprehensively implemented multiple bioinformatic analysis strategies to screen considerable potential key genes for myogenic differentiation and revealed the interesting epigenetic regulation behavior of cooperatively binding transcription factors (TFs) (Figure 1). Robust DEGs were identified and validated by using the RRA method and multiomics data (RNA-Seq, expression arrays, and H3K27ac ChIP-Seq). Then, their biological functions were analyzed, and the core robust differentially expressed TFs (DETFs) were screened. Further use of TF ChIP-Seq data and diverse genome annotations, including H3K27ac signals, super-enhancers (SEs), and conserved DNA elements, revealed the cooperative binding of robust DETFs and their potential for application in labeling key enhancers for myogenesis.

## 2. Results

### 2.1. Published RNA-Seq Datasets during C2C12 Cell Myogenesis Are Poorly Consistent

**Figure 2:**
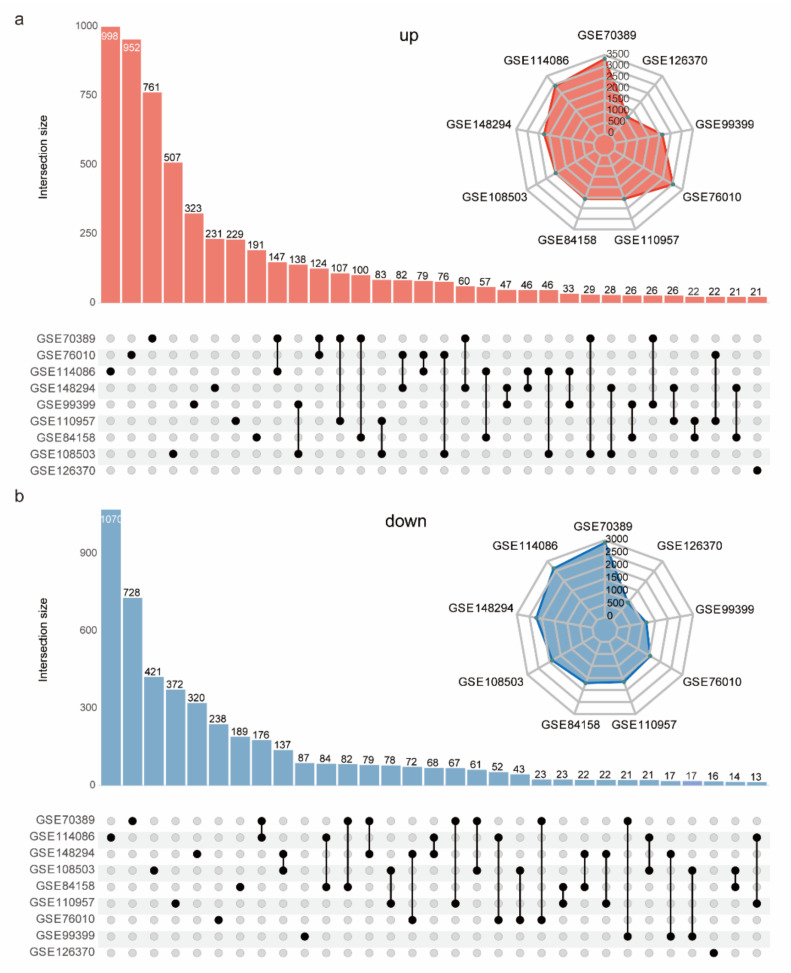
Intersection of DEG lists from 9 RNA-Seq datasets. The number of upregulated (**a**) and downregulated (**b**) genes in the 9 DEG lists (radar plot in the upper-right corner). The respective intersections of upregulated genes (**a**) and downregulated genes (**b**) (multidimensional Venn figure plotted with the ComplexUpset R package).

A total of nine RNA-Seq datasets were collected during C2C12 cell myogenesis, including 21 MB and 21 MT samples (Supplementary Materials) [20,21,22,23,24,25,26,27,28]. Differential expression analysis was implemented for each dataset. When the same identification criteria were used for DEGs (|fold change| > 2, adjusted *p* value < 0.05), the number of DEGs in each dataset varied greatly, ranging from 2003 DEGs (GSE126370) to 6234 DEGs (GSE70389) (Supplementary Materials). The intersecting up- and downregulated DEGs of these datasets were screened out. There were 998, 952, and 761 unique upregulated DEGs in the GSE114086, GSE76010, and GSE70389 datasets, respectively (Figure 2a). There were 1070, 728, and 421 unique downregulated DEGs in the GSE114086, GSE70389, and GSE108503 datasets, respectively (Figure 2b). These results show that the currently published RNA-seq datasets collected during C2C12 cell myogenesis are poorly consistent, and many DEGs will be lost by simply taking the intersecting DEGs. We believe that unavoidable differences in manual operations, experimental platforms, and sequencing platforms, etc., are the main factors causing inconsistency between RNA-Seq datasets.

### 2.2. Considerable Robust DEGs Were Identified by Integrating Nine RNA-Seq Datasets

**Figure 3:**
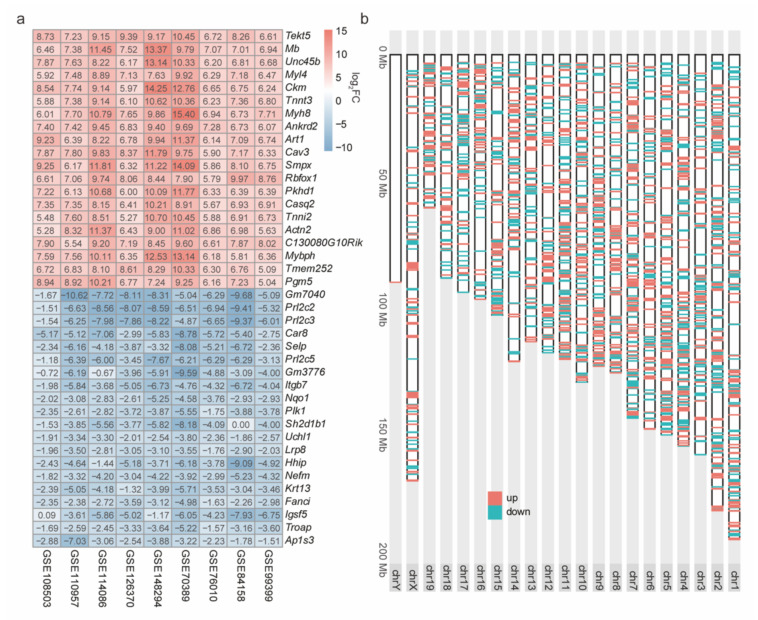
Robust DEGs during C2C12 cell myogenesis were identified using the RRA method. (**a**) Top 20 up- and downregulated robust DEGs across 9 RNA-Seq datasets during C2C12 cell myogenesis. The log_2_FC represent log_2_(fold change) of the gene expression level. (**b**) Genomic localization of the robust DEGs on chromosomes.

The above results have shown that the intersection operation is not an appropriate way to integrate the DEGs of multiple RNA-Seq datasets and cannot take full advantage of the quantity of multiple datasets. To identify robust DEGs in the process of C2C12 cell myogenesis, this study used the RRA method to integrate the above differential expression analysis results for nine C2C12 cell RNA-seq datasets (Figure 3a). A total of 3140 robust DEGs (|fold change| > 2, adjusted *p* value < 0.01) were identified, including 1784 upregulated genes and 1356 downregulated genes (Supplementary Materials). The proteins encoded by the *Mb*, *Myl4*, *Myh8*, and *Mybph* genes, among the top 20 most strongly upregulated genes, are important components of myofibers [34,35,36] (Figure 3a). In addition, the well-known myogenic factor genes (*Myog*, *Myf6*, *Mef2a*, *Mef2b*, *Mef2c*, and *Mef2d*) [3,4,5,6,7] and cell proliferation genes (*Cdk1*, *Cdk4*, and *Cdk6*) [37] showed uniform up- and downregulation trends, respectively (Supplementary Materials). This is consistent with previous knowledge that C2C12 cells escape the cell cycle and differentiate into MTs once stimulated by differentiation induction [38]. Furthermore, we observed that the robust DEGs were distributed almost throughout all chromosomes (Figure 3b), indicating that myogenesis is accompanied by extremely drastic transcriptional regulation behavior, suggesting the necessity of identifying robust DEGs to avoid misinterpretation caused by unstable DEGs.

### 2.3. The Robust DEGs Were Validated by Array Expression Profiles and H3K27ac Signals

**Figure 4:**
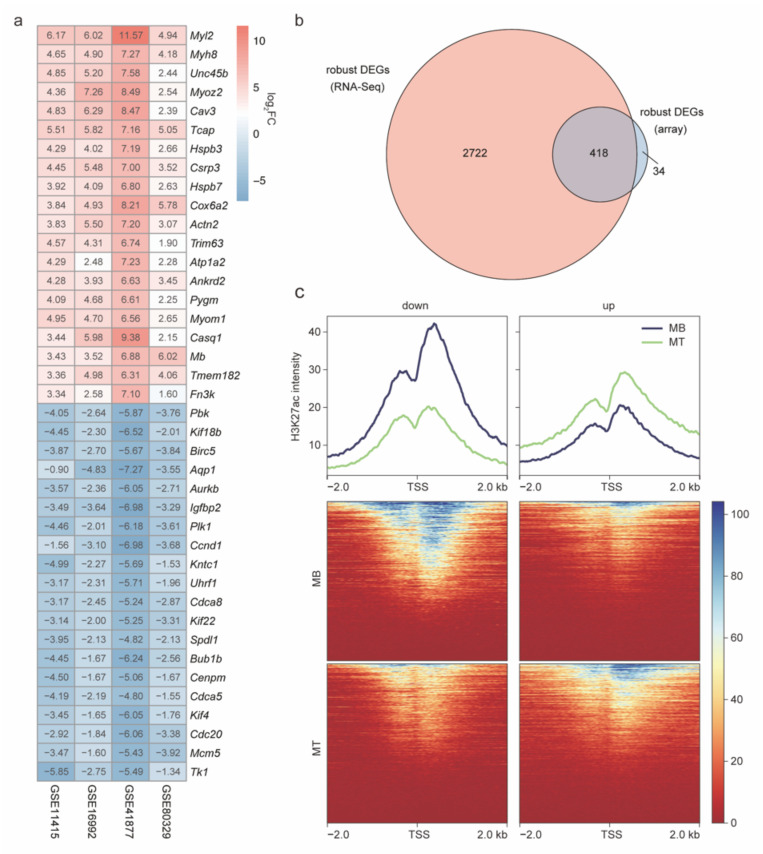
Reliability analysis of robust DEGs. (**a**) Top 20 up- and downregulated robust DEGs across 4 array expression datasets during C2C12 cell myogenesis. The log_2_FC (log_2_ fold change) represent gene expression change. (**b**) The overlap of robust DEGs identified using RNA-Seq datasets and array expression datasets. (**c**) H3K27ac signal levels in the TSSs of up- and downregulated robust DEGs in C2C12 MBs and MTs. The H3K27ac intensity represents read intensity from H3K27ac ChIP-Seq data.

In order to evaluate the accuracy of the robust DEGs identified above, we also analyzed the array expression profile data [11,12,13,14] during C2C12 cell myogenesis and the corresponding H3K27ac ChIP-seq data [28]. The DEGs of the four array datasets were identified and integrated by the RRA method (Figure 4a), and a total of 452 robust DEGs (array) were screened out (|fold change| > 2, adjusted *p* value < 0.01), including 212 upregulated genes and 240 downregulated genes. Among them, 418 robust DEGs (array), approximately 92%, overlapped with the robust DEGs (Figure 4b), indicating that these array expression profiles support the robust DEGs identified using RNA-Seq datasets. In addition, H3K27ac histone modification is a classic promoter and enhancer activation marker that can reflect the transcriptional activation level [39]. We further observed that the transcription start sites (TSSs) of the upregulated robust DEGs exhibited higher H3K27ac signals than those of the downregulated robust DEGs in C2C12 MTs (Figure 4c), indicating that the upregulated robust DEGs were actively epigenetically regulated during myogenesis. The opposite pattern was observed in C2C12 MBs (Figure 4c). These results strongly support the reliability of the robust DEGs identified in this study.

### 2.4. The Functions of Robust DEGs Were Investigated by GO/KEGG Analysis

**Figure 5:**
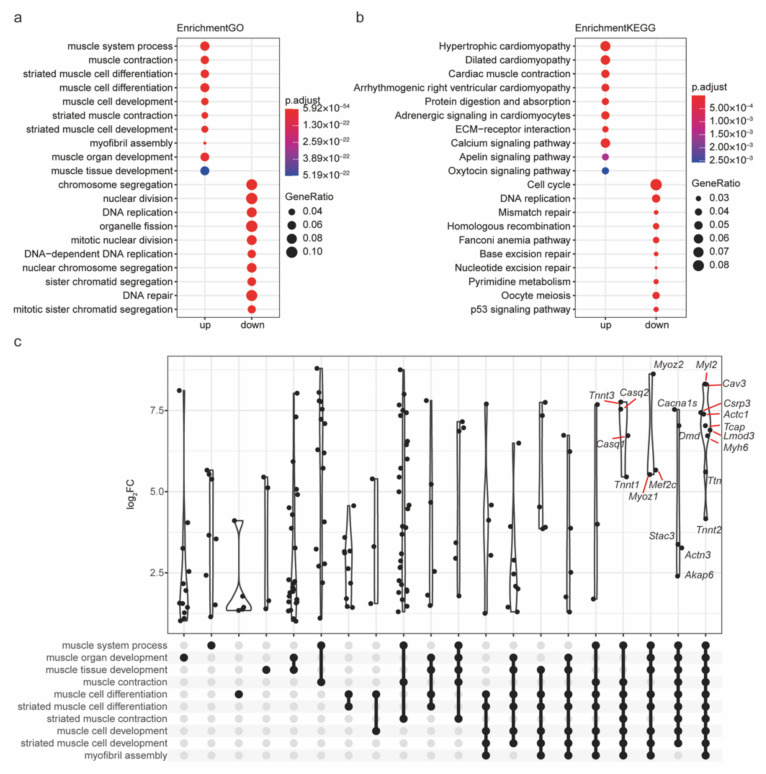
Enrichment analysis of robust DEGs for GO/KEGG terms. (**a**) Top 10 GO biological processes and (**b**) top 10 KEGG pathways enriched for up- and downregulated robust DEGs, respectively. (**c**) The overlapping genes among the GO terms enriched for upregulated robust DEGs. The log_2_FC represent log_2_(fold change) of the gene expression level.

Based on the robust DEGs, the biological processes and cell signaling pathways during myogenesis can be more accurately revealed than ever before [40]. Notably, up- and downregulated robust DEGs were enriched in distinct GO biological processes and KEGG pathways (Figure 5a,b, Supplementary Materials). Regarding GO terms, the upregulated robust DEGs were highly enriched in muscle development-related processes, including muscle cell differentiation, the muscle system process, and muscle organ development. The downregulated robust DEGs were highly enriched in cell division- and proliferation-related processes, including nuclear division, DNA replication, and organelle fission. Regarding KEGG terms, the upregulated robust DEGs were highly enriched in signaling pathways involved in muscle development, including protein digestion and absorption, the ECM-receptor interaction, and the calcium signaling pathway. The downregulated robust DEGs were highly enriched in signaling pathways involved in cell division and proliferation, including the cell cycle, DNA replication, and mismatch repair. The results of GO/KEGG analysis were highly consistent with the biological process that this research focuses on. The overlapping genes among GO terms are often considered to be important communication nodes because they are involved in multiple biological processes. Our study further found that overlapping genes among the GO terms enriched for upregulated robust DEGs, such as *Myl2*, *Cav3*, *Csrp3*, *Actc1*, *Tcap*, *Lmod3*, and *Myh6*, seemed to exhibit more dramatic upregulation (Figure 5c). These results clearly reveal the molecular mechanism of myogenesis and highlight the importance of overlapping genes.

### 2.5. The Core Robust DETFs Were Screened by the PPI Network

**Figure 6:**
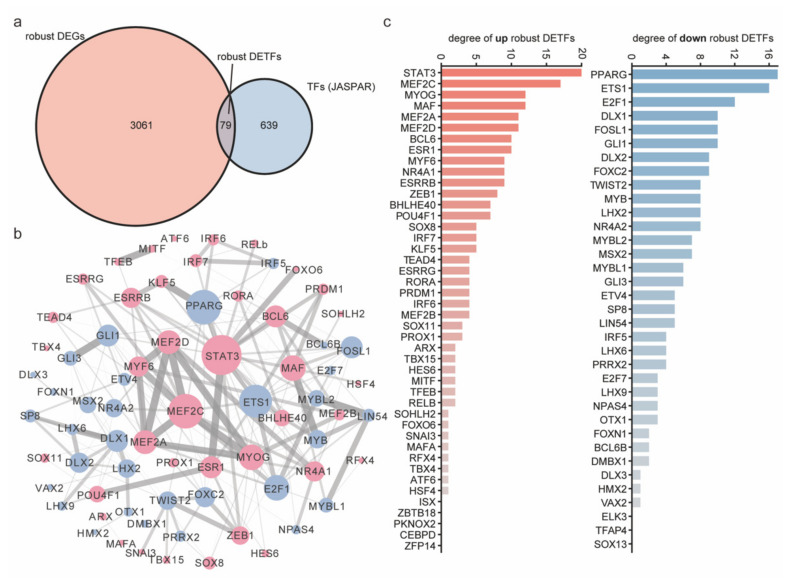
PPI network analysis of robust DETFs. (**a**) The overlap between robust DEGs and vertebrate TFs from the JASPAR database yields robust DETFs. (**b**) PPI network for robust DETFs. Thicker lines with heavier black represent stronger protein interactions. Red represents upregulation, and blue represents downregulation. (**c**) Lists of up- and downregulated robust DETFs sorted by PPI degree.

The advancement of myogenesis is accompanied by drastic fluctuations in the transcriptome, and the TFs are upstream of the transcriptional regulatory network. To explore the core TFs during the myogenesis process, a PPI analysis of robust DETFs was implemented. We downloaded the TF list of vertebrates from the JASPAR database. Then, it was found that robust DEGs contained 79 DETFs (44 upregulated TFs and 35 downregulated TFs) (Figure 6a, Supplementary Materials). Further analysis of PPI network was performed (Figure 6b). Additionally lists of up- and downregulated robust DETFs sorted by PPI degree are presented (Figure 6c). Several known myogenic factors (STAT3, MEF2C, MYOG, MEF2A, MEF2D, MYF6, and NR4A1) [3,4,5,6,7,9,10] and functionally ambiguous factors (MAF, BCL6, and ESR1) were at the core of upregulated robust DETFs. Several factors known to control cell cycling and proliferation (E2F2, MYB, ETS1, FOSL1, and GLI1) [41,42,43,44,45], a myogenic differentiation inhibitor (TWIST2) [46,47], and functionally ambiguous factors (PPARG, DLX1, DLX2, and FOXC2) were at the core of downregulated robust DETFs. In terms of definition, TFs exert regulatory roles by binding to cis-regulatory elements (CREs), such as enhancers. By mining robust DETFs, we observed the dynamic recruitment (upregulation) and decommissioning (downregulation) of TFs. The further discovery of core TFs could help us clarify the main transcriptional regulatory behaviors during myogenesis.

### 2.6. The Cooperative Binding Modes of KLF5 and Three Key Myogenic Factors Were Revealed

**Figure 7:**
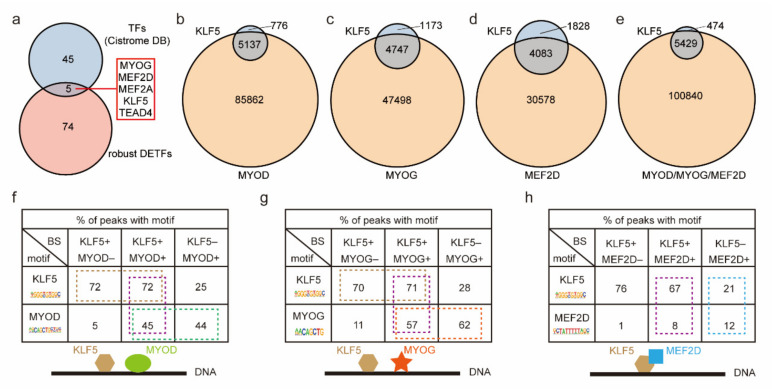
Screening the cooperative binding of robust DETFs. (**a**) The overlap of robust DETFs and TFs of C2C12 cells from the Cistrome DB. The KLF5 peaks overlap with the MYOD (**b**), MYOG (**c**), and MEF2D (**d**) peaks. (**e**) MYOD, MYOG, and MEF2D peaks were merged and intersected with KLF5 peaks. Because of the processing of merged peaks, the number of KLF5 peaks fluctuates slightly within each group. (**f**) The proportion of peaks harboring KLF5 or MYOD motifs among the overlapping and nonoverlapping peaks of KLF5 and MYOD. (**g**) The proportion of peaks harboring KLF5 or MYOG motifs among the overlapping and nonoverlapping peaks of KLF5 and MYOG. (**h**) The proportion of peaks harboring KLF5 or MEF2D motifs among the overlapping and nonoverlapping peaks of KLF5 and MEF2D.

The cooperative binding of TFs to enhancers to increase the expression level of target genes is an important method of epigenetic regulation [48]. With the development of next-generation sequencing (NGS) technology, ChIP-Seq data have grown exponentially. Taking full advantage of these data will be greatly beneficial for discovering the mechanism of cooperation between TFs. We first downloaded the peak bed files (containing binding site information) of TF ChIP-seq data collected and curated by the Cistrome DB, including 50 TFs from C2C12 cells. Among them, MYOG, MEF2D, MEF2A, KLF5, and TEAD4 were found to be robust DETFs (Figure 7a). We further observed that in C2C12 MTs, the KLF5-binding sites were highly overlapping (86%, 80%, and 69%) with the binding sites of three key myogenic factors (MYOD, MYOG, and MEF2D) (Figure 7b–d), and 93% of the KLF5-binding sites coincided with the binding sites of at least one of these three myogenic factors (Figure 7e). These results strongly suggest that KLF5 cooperatively binds with these three myogenic factors.

Indeed, referring to previous methods [38], we observed that among KLF5+/MYOD+ peaks (cobinding sites of KLF5 and MYOD), 72% and 45% harbored KLF5 and MYOD motifs, respectively (Figure 7f). Interestingly, stable ratios were further observed in other types of peaks. Seventy-five percent of KLF5+/MYOD- peaks (non-MYOD KLF5-binding sites) harbored KLF5 motifs, and 44% of KLF5-/MYOD+ peaks (non-KLF5 MYOD-binding sites) harbored MYOD motifs. We speculated that KLF5 and MYOD rely on their own motifs, but not on each other’s motifs, to coordinately bind to DNA sequences. Similar results were observed (Figure 7g), and we inferred that KLF5 and MYOG also coordinately bind in an independent manner. Among the KLF5+/MEF2D+ peaks, 67% harbored KLF5 motifs, and only 8% harbored MEF2D motifs (Figure 7h). However, 21% and 12% of KLF5-/MEF2D+ peaks, which were relatively comparable, harbored KLF5 and MEF2D motifs, respectively. Altogether, the above findings suggest that MEF2D relies on KLF5 motifs to coordinately bind to DNA sequences.

### 2.7. KLF5-Binding Sites Could Mark Key Enhancers for Myogenesis

**Figure 8:**
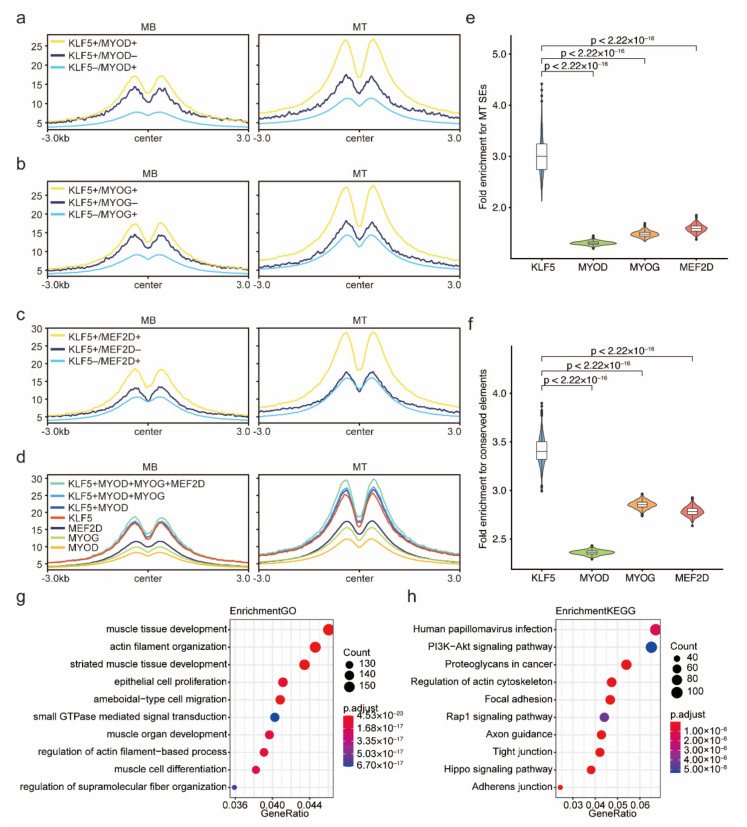
Enhancer properties of the KLF5-binding site for myogenesis. In C2C12 MBs and MTs, H3K27ac intensity of overlapping and nonoverlapping peaks in 3 groups: KLF5/MYOD (**a**), KLF5/MYOG (**b**), and KLF5/MEF2D (**c**). (**d**) In C2C12 MBs and MTs, H3K27ac intensity of 7 types of peaks: MYOD peaks, MYOG peaks, MEF2D peaks, KLF5 peaks, overlapping peaks of KLF5 and MYOD, overlapping peaks of KLF5, MYOD, and MYOG, overlapping peaks of KLF5, MYOD, MYOG, and MEF2D. Fold enrichments of KLF5-, MYOD-, MYOG-, and MEF2D-binding sites for SEs from C2C12 MTs (**e**) and mammalian-conserved elements from genomic evolutionary rate profiling (GERP) (**f**). Enriched GO terms (**g**) and KEGG pathways (**h**) for the KLF5-binding sites specific to C2C12 MTs relative to MBs.

As hubs of TFs, enhancers are classically marked by H3K27ac modification signals by orderly recruiting of TFs to advance biological processes [39]. Importantly, the peak H3K27ac intensity of the three comparable groups was KLF5+/MYOD+ > KLF5+/MYOD– > KL5–/MYOD+, KLF5+/MYOG+ > KLF5+/MYOG– > KL5–/MYOG+, and KLF5+/MEF2D+ > KLF5+/MEF2D– > KL5–/MEF2D+ (Figure 8a–c). In addition, the peak H3K27ac intensity of the four TFs was KLF5 > MEF2D > MYOG > MYOD (Figure 8d). These results indicate that the cooperative binding of KLF5 increases H3K27ac levels at the binding sites of these three myogenic factors. Previous research revealed that MYOD, MYOG, and MEF2D sequentially play a role in myogenesis [38,49,50,51]. We tried to simulate the assembly process of the enhancer transcription machinery during myogenesis. On the basis of the KLF5-binding signal, MYOD-, MYOG-, and MEF2D-binding signals were sequentially superimposed, and a gradually rising H3K27ac intensity was observed (Figure 8d). This result suggests that more fully cooperative binding of TFs means higher transcriptional regulation ability. SEs, a class of key enhancer clusters, have a stronger ability to enrich TFs and regulate target gene expression than conventional enhancers and determine the expression of cell identity genes [52,53,54]. We downloaded Mt SEs from previous reports [38] and further found that the KLF5-binding sites have a greater ability to enrich Mt SEs than the MYOD-, MYOG-, and MEF2D-binding sites (Figure 8e). We also found that the KLF5-binding sites showed higher enrichment for conserved DNA sequence elements from the Ensembl database than the MYOD-, MYOG-, and MEF2D-binding sites (Figure 8f). According to the above results, we suggest that the KLF5-binding sites have the potential to label key enhancers of myogenesis, and this feature may occur across mammalian species. In addition, we annotated the KLF5-binding sites specific to C2C12 MTs to reveal the function of KLF5 on myogenesis, referring to the Genomic Regions Enrichment of Annotations Tool (GREAT) [55]. The results show that the KLF5-binding sites are significantly enriched in myogenesis-related GO terms (muscle tissue development, muscle organ development, and muscle cell differentiation) and KEGG pathways (PI3k-AKt signaling pathway [56,57], Rap1 signaling pathway [56], and Hippo signaling pathway [58,59]) (Figure 8g,h, Supplementary Materials), suggesting that KLF5 may regulate myogenesis through these three pathways. This result supports the potential application of KLF5-binding sites to label key enhancers of myogenesis.

## 3. Discussion

The basis of this study was the identification of robust DEGs. A previous study identified 824 consistent DEGs across three datasets during myogenic differentiation by taking intersections [40]. However, because of batch effects, this approach unavoidably loses many real DEGs due to taking intersections, and the more integrated datasets there are, the less consistent the DEGs. Our results confirm this (Figure 2a,b). The RRA algorithm can assign a significant *p* value to each DEG to quantify its confidence so that more high-throughput expression datasets can present greater power and confidence to identify robust DEGs. With the geometric growth of high-throughput gene expression datasets, the advantages of the RRA method become more apparent. The more datasets that were integrated, the more robust DEGs were identified by the RRA method. Our results confirmed this, with the RRA method yielding 3140 (nine RNA-Seq datasets) and 418 (four array expression datasets) robust DEGs. The high coincidence of the results of these two types of high-throughput expression datasets and the adaptation of H3K27ac intensity fluctuations around TSSs fully demonstrate the validity of the robust DEGs identified in this project.

We further screened key genes among the robust DEGs. Based on the robust DEGs not previously reported, detailed and accurate enriched GO/KEGG terms are presented, which are very helpful for understanding the cellular and molecular mechanisms of myogenesis. Overlapping genes for these annotated terms often receive more attention because of their junction locations. An interesting general phenomenon was observed: the upregulated robust DEGs associated with multiple GO terms showed dramatic upregulation. This may be because they play multiple roles in relation to different terms and thus require high expression abundance. The “cross-talk” of these GO terms includes shared components, protein interactions, and stimulators. The perturbation of overlapping genes could have synergistic effects on certain biological processes. We speculate that these overlapping genes are vital for muscle development, with myogenesis as a core step. Indeed, among them, existing studies have shown that mutations in the myosin regulatory light chain 2 (MYL2) [60] and caveolin 3 (CAV3) [61] genes cause severe human muscle disease. In addition, a SNP mutation in the porcine cysteine- and glycine-rich protein 3 (CSRP3) gene was closely related to pig meat quality [62]. Further exploration of the role of these overlapping genes would be well worthwhile.

Moreover, given that TFs are upstream regulatory components in transcriptional regulatory networks, we also screened robust DETFs. PPI network analysis was performed to further select core TFs. Among the top 10 high-degree nodes of upregulated robust DETFs, seven TFs reported positive myogenic factors. The functions of the remaining three TFs, including muscle aponeurosis fibromatosis (MAF), B-cell lymphoma 6 (BCL6), and estrogen receptor 1 (ESR1), in myogenesis or muscle development are poorly reported. The *Maf* gene is closely related to the process of cell differentiation, and the E-box element of its promoter has high affinity with MYOD [63]. The *Bcl6* gene prevents apoptosis during myocyte differentiation [64] and is active during myocyte terminal differentiation due to a specifically elevated expression level and an open chromatin signal strength [65]. The C allele of the genetic polymorphism rs2234693 in the human *ESR1* gene was found to prevent muscle damage by reducing muscle stiffness [66]. Research on them may yield seminal results in myogenesis. We then used multiple means to reveal the cooperative binding behavior of robust DETFs and analyzed the enhancer activity characteristics of their binding sites. ChIP-Seq peaks of TFs annotated their binding sites. A previous report has confirmed that the binding sites of KLF5 overlap with that of MYOD and MEF2D [8]. We further performed pairwise intersections of large-scale ChIP-Seq peaks from C2C12 cells and screened out that the binding sites of KLF5 and MYOG are also highly overlapping. Not only that, we scrutinized their cooperative binding modes. Both MYOD and MYOG bind to DNA sequences in a KLF5-independent manner, while MEF2D binding is KLF5-dependent. This result was not previously reported.

MRFs and MEF2s dominate the myogenic differentiation program. Among them, MYOD, the master TF, is responsible for activating genes expressed in the early stage of myogenic differentiation and has been shown to be critical for enhancer assembly and activation [38]. Because of the recognition of E-box DNA sequences, MYOD and MYOG coregulate several muscle-specific genes [50,67]. However, their functions are distinct. MYOD first induces chromatin modification, and then MYOG binds to activate gene expression [50,51]. MEF2D lacks independent myogenic activity and requires cooperation with MRF family members to activate the myogenic differentiation program [68]. These three TFs are indeed important, but their binding sites (~100,000) are particularly widespread throughout the genome of C2C12 cells. This is not conducive to screening out key enhancers. In contrast, KLF5-binding sites (~6000) are relatively rare. Our study shows that cooperative binding of KLF5 increases H3K27ac levels at the binding sites of these three myogenic factors. Consistent with this, the enhancers (~36,000) for myogenesis in bovine satellite cells were enriched with KLF5-binding sites [69]. These results suggest that KLF5-binding signal is a marker of enhancers for myogenesis. Indeed, further investigation showed that KLF5-binding sites are highly enriched in MT SEs and are highly conserved compared to MYOD-, MYOG-, and MEF2D-binding sites. Therefore, it was concluded that KLF5-binding sites could be exploited to filter redundant MYOD-, MYOG-, and MEF2D-binding sites to focus on key enhancers for myogenesis. Consistent with this, our study showed that the KLF5-binding sites specific to C2C12 MTs were annotated to be closely related to myogenesis processes. Additionally, KLF5 may regulate myogenesis through the PI3K-AKt signaling pathway, Rap1 signaling pathway, and the Hippo signaling pathway. Moreover, in C2C12 MTs, we found that KLF5 binds to the regulatory regions (5 kb upstream and 1 kb downstream of TSSs) of TEAD1/3/4, which are components of the Hippo signaling pathway (Supplementary Materials) and are required for myogenic differentiation [70]. Among them, TEAD4 has been confirmed to be the downstream target gene of MYOD and MYOG. Given that KLF5 cooperatively binds with MYOD, MYOG, and MEF2D, these results imply that MYOD, MYOG, MEF2D, and KLF5 cooperatively regulate TEAD factors during myogenesis. Taken together, we believe that KLF5-binding sites have potential applications in labeling key enhancers of myogenic differentiation. This finding may have intermediate applicability in mammals.

## 4. Materials and Methods

### 4.1. Dataset Collection

This study aimed to investigate DEGs during myogenic differentiation of C2C12 cells. To take full advantage of the published datasets, we only focus on robust DEGs during the state switch of wild-type C2C12 cells from MBs and MTs, regardless of differentiation time and differentiation medium composition. The datasets across C2C12 cell myogenesis used in this study are from the GEO database (https://www.ncbi.nlm.nih.gov/geo/, accessed on 25 April 2022), including 9 RNA-Seq datasets (21 each for MB and MT samples), 4 expression array datasets (12 each for MB and MT samples), and 1 H3K27ac ChIP-Seq dataset (1 each for MB and MT samples). Detailed information, including dataset number, sample number, sequencing platform, differentiation stage, author, and publication year, is listed in the Supplementary Materials.

### 4.2. DEG Identification and RRA Integration

For the RNA-Seq datasets, the mouse reference genome was obtained from the Ensembl database. Available online: http://ftp.ensembl.org/pub/release-99/fasta/mus_musculus/dna/Mus_musculus.GRCm38.dna.primary_assembly.fa.gz (accessed on 25 April 2022). The gene annotation information also obtained from the Ensembl database. Available online: http://ftp.ensembl.org/pub/release-99/gtf/mus_musculus/Mus_musculus.GRCm38.99.chr.gtf.gz (accessed on 25 April 2022). After removing low-quality reads and adapter sequences, the remaining reads in the fastq files were aligned to the genome using HISAT2 software [71]. The gene expression abundance was quantified with FeatureCounts software [72]. DEGs were identified using the DESeq2 R package [73]. For the array datasets, DEGs were identified using the limma R package [74]. Multiple DEG lists were first sorted by log2FC of expression values and then integrated with the RobustRankAggreg R package [30] to identify robust DEGs.

### 4.3. ChIP-Seq Data Analysis

For the H3K27ac ChIP-Seq dataset, the reads from the fastq files were aligned to the mouse genome using Bowtie2 software [75]. Reads with low-quality alignments were filtered using SAMtools software [76]. Duplicate reads were eliminated with Sambamba software [77]. The bamCoverage tool of deepTools software [78,79] was used to convert the alignment files in bam format to bigWig files. The plotHeatmap tool of deepTools software was used to draw the genome interval profile.

### 4.4. GO/KEGG Analysis and PPI Network Construction

Enrichment analysis of GO terms and KEGG pathways was performed on the gene sets using the ClusterProfiler R package [80]. Overlapping genes for multiple target terms were extracted using the ggupset R package. Available online: https://github.com/const-ae/ggupset (accessed on 25 April 2022). The STRING database was used to download the protein interaction relationship file of the target gene set. Available online: https://string-db.org/ (accessed on 25 April 2022). The PPI network was constructed and drawn with Cytoscape software and Adobe Illustrator software. Available online: https://cytoscape.org/ (accessed on 25 April 2022), https://www.adobe.com/products/illustrator.html (accessed on 25 April 2022)

### 4.5. Cooperative Binding Analysis of TFs

Peak bed files of TF ChIP-Seq data were downloaded from the Cistrome DB database. Available online: http://cistrome.org/db/#/ (accessed on 25 April 2022). Peaks on non-standard chromosomes were removed first. Then, the intersect tool of BEDTools software [81,82] was used to investigate the overlap of peaks of different TFs and to further screen out the TFs with overlapping peaks accounting for more than 50%. The findmotifsgenome.pl tool of homer software was used to scan genomic regions for TF motifs. The H3K27ac profiles of overlapping and nonoverlapping peaks were plotted using the plotProfile tool of deepTools software.

### 4.6. Enrichment for MT SEs of Genomic Regions

The genomic localization information of MT SEs was obtained from previous reports [38]. MT SEs that overlapped with target genomic regions were extracted using the BEDTools intersect tool. Artificial interval sets were randomly generated by simulating MT SEs using the BEDTools shuffle tool and repeated 1000 times. We then calculated the number of MT SEs that overlapped with the target interval set relative to the number of MT SEs that overlapped with the artificial interval sets to obtain the fold enrichment for MT SEs in the target interval set. The Student’s two-tailed *t*-test was performed in R environment to compare the fold enrichments for MT SEs between two genomic interval sets. Statistical significance level was set to *p*-value < 0.05.

### 4.7. Conservative Analysis of Genomic Regions

The bigBed file of conserved DNA elements in mouse was obtained from the Ensembl database. Available online: http://ftp.ensembl.org/pub/release-100/bed/ensembl-compara/103_mammals.gerp_constrained_element/gerp_constrained_elements.mus_mumuscul.bb (accessed on 25 April 2022). The file then was converted into a bed file with bigBedToBed software [83]. Using the above method, the fold enrichment for conserved elements in the target interval set was calculated. The Student’s two-tailed *t*-test was performed in R environment to compare the fold enrichments for conserved elements between two genomic interval sets.

### 4.8. Functional Annotation of Genomic Regions

In this study, the KLF5-binding sites were associated with genes with reference to the CREAT principle. First, each gene was assigned a gene regulatory domain (5 kb upstream and 1 kb downstream of TSSs). Then, use the BEDTools intersect tool to associate KLF5-binding sites with genes once the KLF5-binding sites overlap with the corresponding regulatory regions. The gene regulatory region extends up to 1000 kb in both directions until a KLF5-binding site falls. Finally, enrichment for GO terms and KEGG pathways was performed on genes associated with KLF5-binding sites using the ClusterProfiler R package.

## 5. Conclusions

We used multiomics data to identify and validate 3140 robust DEGs, including 79 robust DETFs, during myogenic differentiation. The 44 upregulated robust DETFs may contain some novel myogenic factors, among which MAF, BCL6, and ESR1 are of particular interest due to their high degree of PPI network connectivity. We also extended previous knowledge on the regulatory features of KLF5, an upregulated robust DETF, in myogenic differentiation. The KLF5-binding signal is recognized as a potential key enhancer marker for myogenesis. Future studies may be convenient to dissect the core regulatory mechanism of myogenesis relying on the KLF5-binding signal.

## Data Availability

The sequence data presented in this study are openly available in GEO database. Available online: https://www.ncbi.nlm.nih.gov/geo/ (accessed on 25 April 2022).

## Supplementary Materials

The following supporting information can be downloaded at: https://www.mdpi.com/article/10.3390/ijms23116002/s1.
